# How Altered Ribosome Production Can Cause or Contribute to Human Disease: The Spectrum of Ribosomopathies

**DOI:** 10.3390/cells9102300

**Published:** 2020-10-15

**Authors:** Giulia Venturi, Lorenzo Montanaro

**Affiliations:** 1 Department of Experimental, Diagnostic and Specialty Medicine, Alma Mater Studiorum-University of Bologna, Via Massarenti 9, 40138 Bologna, Italy; giulia.venturi13@unibo.it; 2 Center for Applied Biomedical Research, Alma Mater Studiorum-University of Bologna, Via Massarenti 9, 40138 Bologna, Italy; 3 Azienda Ospedaliero-Universitaria di Bologna, Via Albertoni 15, 40138 Bologna, Italy

**Keywords:** ribosome biogenesis, rare diseases, Diamond Blackfan anemia, X-linked dyskeratosis congenita, 5q− syndrome, Shwachman-Diamond syndrome, Treacher Collins syndrome, cartilage hair hypoplasia, cancer

## Abstract

A number of different defects in the process of ribosome production can lead to a diversified spectrum of disorders that are collectively identified as ribosomopathies. The specific factors involved may either play a role only in ribosome biogenesis or have additional extra-ribosomal functions, making it difficult to ascribe the pathogenesis of the disease specifically to an altered ribosome biogenesis, even if the latter is clearly affected. We reviewed the available literature in the field from this point of view with the aim of distinguishing, among ribosomopathies, the ones due to specific alterations in the process of ribosome production from those characterized by a multifactorial pathogenesis.

## 1. Introduction

Specific defects in the process of ribosome production lead to a heterogeneous group of human disorders that are well known today as ribosomopathies [1,2]. The term ribosomopathy, with its correspondent meaning, was first suggested [3] for the skin and bone marrow failure syndrome X-linked dyskeratosis congenita (X-DC), in which the human pseudouridine synthase dyskerin is mutated [4]. Among its functions, dyskerin mediates the modification of approximately 100 specific uridine residues to pseudouridines in rRNA, an essential step of ribosomal biogenesis. Soon after, it was discovered that approximately one fourth of Diamond Blackfan anemia (DBA) patients harbor a mutation in the gene encoding the ribosomal protein (RP) S19 (or eS19 according to the new nomenclature [5]) [6], suggesting that the term ribosomopathy could be shared by more than a single disease. The list of recognized ribosomopathies then grew rapidly to include Schwachman-Diamond syndrome (SDS), cartilage hair hypoplasia (CHH), and Treacher Collins syndrome (TCS) [7,8,9]. Ever since the earliest classification attempts, these five disorders have been considered examples of known or suspected inherited ribosomopathies [10]. Their number continued to grow further, coming to include a list of other less characterized inherited disorders, as well as acquired conditions such as the 5q− myelodysplastic syndrome [11] and cancer [12,13]. To understand the molecular mechanism underlying most of these disorders, it may be helpful to quickly review the fundamental steps in ribosome production in human cells.

Ribosomes are ribonucleoprotein complexes dedicated to messenger RNA translation and protein synthesis. Human cytoplasmic ribosomes are made of four ribosomal RNA molecules and approximately 80 proteins divided into two subunits: the large subunit (60S) and the small subunit (40S). The first accounts for three rRNAs, 5S, 5.8S, and 28S together with 47 ribosomal proteins (RPs). The small subunit is made up of 18S rRNA and 33 RPs.

**Figure 1:**
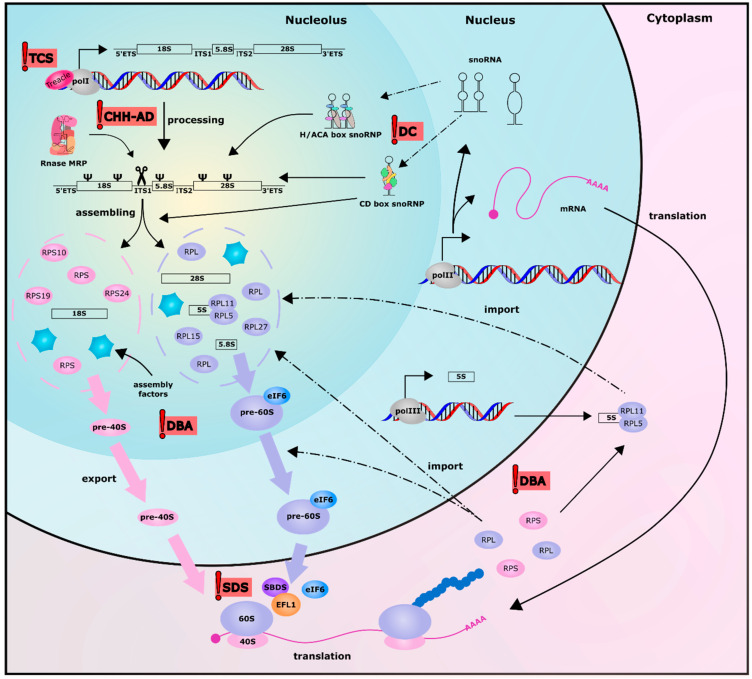
Schematic representation of the ribosome biogenesis process in human cells. The red flags highlight the steps where mutations in genes encoding for ribosomal proteins or for factors involved in ribosome biogenesis give rise to the five ribosomopathies originally identified: Treacher Collins syndrome (TCS), X-linked dyskeratosis congenita, cartilage hair hypoplasia-anauxetic dysplasia (CHH-AD), Diamond Blackfan anemia (DBA) and Schwachman-Diamond syndrome (SDS).

Ribosome biogenesis is an intricate and coordinated process (reviewed in [14,15]) that occurs in the nucleolus and later in the cytoplasm (Figure 1); it involves more than 200 trans-acting factors [16] that are required during the numerous steps of ribosomal subunits maturation. All three RNA polymerases are required in this process: RNA polymerase I is needed for the transcription of 28S, 5.8S, and 18S rRNAs [17], RNA pol II produces the mRNAs of the 80 ribosomal proteins and numerous ribosomal processing factors, and RNA pol III synthetizes 5S rRNA [18]. In the nucleolus, RNA polymerase I, complexed with different transcription initiation factors, synthetizes a large polycistronic transcript (47S) from the rDNA genes present in hundreds of copies within the cell [19]. The pre-rRNA 47S obtained is made up of two external transcribed spacers (ETS) positioned at 5’ and 3′ of the molecule, while the sequence of 18S, 5.8S, and 28S are separated by two internal transcribed spacers (ITS1 and ITS2).

The maturation of this polycistronic transcript occurs in the nucleolus and starts with the action of a series of small nucleolar ribonucleoprotein complexes, namely C/D and H/ACA box snoRNPs [20]. C/D box snoRNPs are made up of fibrillarin and accessory proteins such as Nop56, Nop58, 15.5K/NHPX, and the small nucleolar RNAs (snoRNA) characterized by box C and D. This complex, guided by the complementary hybridization with the sequence of the snoRNA, catalyzes the site-specific 2′-*O*-methylation of the ribose of rRNA [21]. Moreover, the complex is also involved in rRNA processing and folding [20]. Recently, a new class of specialized C/D box snoRNA, which are able to guide cytosine rRNA acetylation has been reported [21]. On the other hand, the H/ACA snoRNP complex is made up of dyskerin, Nhp2, Nop10 and Gar1, and the H/ACA snoRNA component. This complex acts similarly to the C/D box snoRNPs: the H/ACA box snoRNA guides the complex to a specific uridine in rRNA, while dyskerin catalyzes the conversion of this uridine into pseudouridine [22]. These modifications can be found in functionally important regions of the ribosome, some of them being essential to regulate translational efficiency [23] and fidelity [24]. In addition, numerous C/D boxes or H/ACA boxes snoRNA not involved in ribose 2′-*O*-methylation or in rRNA pseudouridylation, like U3, U8, U14, U22, U17, and some long non-coding RNAs like RMRP are involved in rRNA processing and maturation [25].

The ribosomal RNA also undergoes a series of processes and assembly events that give rise to ribosome subunits. Some alternative processing pathways are described, although the most common one starts with the cleavage at the 5′ETS end (at the so-called site 01), removal of 3′ETS and, subsequentially, cleavage at site 2 of ITS1 (as extensively described in [15]). This last cleavage by RNAse MRP has the important function of separating the maturation of the two ribosome subunits: the small subunit containing 18S rRNA, and the large subunit containing 5.8S and 28S rRNA. After exonucleolytic and endonucleolytic cleavages at the 3′ of pre-18S rRNA, this RNA and the ribosomal proteins of the small subunit constituting pre-40S particles are exported to the cytoplasm to complete their maturation. On the other hand, the maturation of the large subunit rRNA continues in the nucleolus. It has been reported that, in mammals, two different forms of 5.8S are present: a short (5.8Ss) and a long (5.8Sl) form both originating after the cleavage at site 2 of ITS1 operated by RNase MRP, as is the case with yeast. However, the alternative pathway leading to the formation of 5.8Sl is still unclear, while the trimming of the 5′ end of 5.8S sequence operated by XRN2 leads to the formation of the short form. A second endonucleolytic cleavage occurs in ITS2 and leads to the maturation of 28S rRNA. After the cytoplasmic assembly and nucleolar import of 5S RNP, pre-60S subunits also containing ribosomal proteins can be exported to the cytoplasm to fully complete their maturation process. After this important step, the missing ribosomal proteins are added to the two subunits while completing their maturation [26]. The activation for translation of the nascent 60S subunit occurs when the anti-association factor eIF6 is removed from the large subunit thanks to the activity of EFL1 (elongation factor-like GTPase1) and its cofactor SBDS (Shwachman Bodian Diamond Syndrome) [27]. The correct dissociation of the different assembly factors from both 60S and 40S subunits consist of the final structural quality control and allows the formation of the complete ribosome in presence of a messenger RNA and the translation initiation complex [27,28].

A few years ago, in an attempt to provide a definition, De Keersmaecker, Sulima and Dinman suggested that a ribosomopathy is “any disease associated with a mutation in a ribosomal protein or biogenesis factor impairing ribosome biogenesis in which a defect in ribosome biogenesis or function can be clearly linked to disease causality” [29]. The intention of the Authors was to provide a conservative definition that would not include disorders in which ribosomal defects were not causative. Still, this definition, in addition to “pure” ribosomal disorders, leaves room for disorders in which the defect in ribosome biogenesis only concurs to the pathophysiology of the disease. According to this definition, ribosomopathies can be further classified as disorders whose pathogenesis could be fully ascribed to the defect in ribosome biogenesis and/or ribosomal functions on the one hand, and disorders deriving from defects in multiple cellular functions including ribosome biogenesis on the other. Table 1 shows examples of ribosomopathies following this classification.

## 2. Pure Ribosomopathies

### 2.1. Diamond Blackfan Anemia

Diamond Black anemia (DBA) is frequently present in early childhood as a red cell failure, defined by macrocytic anemia with low reticulocytes and decreased red cell precursors in the bone marrow [51]. Patients may also display a series of skeletal abnormalities and cardiac and genitourinary malformations, together with an increased cancer susceptibility. A very large proportion of patients diagnosed with DBA harbors heterozygous mutations of genes encoding for ribosomal proteins either of the small ribosomal subunit or of the large ribosomal subunit (see Table 1) [33]. The presence of mutated ribosomal proteins could impair ribosome biogenesis at different levels during ribosome assembly, this depends on the single ribosomal protein and the step in which it is involved. In general, these mutations lead to haploinsufficiency for ribosomal protein function, affecting the maturation of the ribosomal subunit containing the protein and ultimately reducing the amount of available functional 80S ribosomes within the cells [52,53,54]. In red cell precursors, this lack of ribosomes impinges the translation of mRNAs encoding key regulators of erythropoiesis such as, for instance, GATA1 [52]. The definition of the pathogenetic mechanisms underlying DBA clearly identifies this disorder as a pure ribosomopathy, in which the defect in a ribosome component clearly causes a misfunction, which can be considered responsible for most of the clinical features of the disease.

Interestingly, a few ribosomal proteins involved in DBA also have extra-ribosomal functions, in addition to their function in ribosome biogenesis. This is the case of RPL11 and RPL5 which play an important role in p53 stabilization [55,56,57,58] and appear to be mutated in approximately 40% of all DBA cases [32,59]. The presentation and severity of the anemia observed in these patients is not clearly different from that due to mutations in genes encoding other ribosomal proteins, confirming that this aspect can be ascribed to the ribosomal defect. Skeletal abnormalities, however, are more frequently associated with RPL5 and RPL11 mutations, which may suggest that the extra-ribosomal functions of these proteins may be involved [32,59] in these traits. On the other hand, skeletal development is known to require high protein synthesis. Therefore, mutations in RPL5 and RPL11 could provoke a severe impairment of ribosome biogenesis such as to cause the skeletal abnormalities associated with these mutations.

### 2.2. Shwachman-Diamond Syndrome

Shwachman-Diamond syndrome (SDS) is a rare autosomal recessive disease characterized by bone marrow failure and multiple developmental abnormalities, such as short stature, skeletal dysplasia, and cognitive impairment. Patients diagnosed with SDS can present an increased risk of transformation to myelodysplastic syndrome (MDS) or acute myeloid leukemia (AML) accurately reviewed in [60,61]. The disease was first identified by Shwachman, Bodian and Diamond in 1964 [62]. In 2003 it was reported that the biallelic mutation of the Shwachman Bodian Diamond Syndrome (*SBDS*) gene is the molecular cause for SDS [7]. The protein encoded by this gene is reported to be a cofactor of the elongation factor-like GTPase1 (EFL1). In the final step of the cytoplasmic ribosome maturation, EFL1 removes the anti-association factor eIF6 from the large ribosomal subunit, thus permitting the entry of 60S subunit in the actively translating pool and in the end, the association with the small ribosomal subunit and the formation of an active ribosome [63]. Therefore, mutations in the SBDS gene can impair ribosome assembly, indicating that SDS may be classified as a ribosomopathy. Recently, mutations in other genes associated with SBDS have been reported to cause SDS-like diseases. In particular, these genes are *DNAJC21*, *EFL1*, and *SRP54*, which are all involved, together with SBDS, in the removal of eIF6 from the ribosome large subunit [37,38,39].

As we have seen, mutations in SBDS and related genes cause reduced ribosome assembly, which, similarly to what occurs in DBA, could affect the global translation but also reduce the tissue-specific translation of selected mRNAs contributing to the development of the disease. This could be the case of highly proliferative tissues in embryonic development. Moreover, the impaired ribosome maturation can induce the activation of the ribosomal stress pathway and p53 stabilization, resulting in different tissue-specific outcomes. For example, loss of the *Sbds* gene in murine pancreas induced p53 activation and loss of digestive enzymes, resulting in an atrophy of post-natal acinar cells due to the induction of cell senescence [64].

### 2.3. Treacher Collins Syndrome

Treacher Collins syndrome (TCS) is an extremely rare congenital disease occurring in 1 out of 50,000 live births. It presents as an autosomal dominant disorder and is characterized by severe craniofacial defects. The symptoms include hypoplasia of the jaw and cheek bones, downward slant of palpebral fissures, cleft palate, and deformity of the ear [9]. The first gene reported to be responsible when mutated for the development of TCS was *TCOF1*, encoding for the protein Treacle. Treacle is a nucleolar protein that co-localizes with the Upstream Binding Factor (UBF) and Pol I and is involved in the transcription of ribosomal DNA [65]. All the different types of mutations reported result in the expression of a truncated protein. However, no correlations between genotype and severity of the disease have been reported [40]. In addition, in 2011, mutations in two genes encoding for Pol I subunits (*POLR1D* and *POLR1C*) were found mutated in a small number of TCS patients [41]. More recently, the work of Sanchez et al. [42] reported mutations connected with TCS also in the *POLR1B* gene. Therefore, all these genes are involved in ribosome biogenesis, and especially in rRNA transcription, so Treacher Collins syndrome may be rightly classified as a pure ribosomopathy. With the help of mice and zebrafish carrying mutations for the genes involved, researchers showed that deficient ribosome biogenesis caused a reduced proliferation of the progenitors of the craniofacial skeleton cells, called neural crest cells (NCC) [42,66,67,68]. It is well known that impaired ribosome biogenesis triggers nucleolar stress with the stabilization of p53 and consequent apoptosis. Therefore, the reduced proliferation and apoptosis of NCC could be caused by nucleolar stress, as demonstrated by the improvement of symptoms in *Tcof1*^+/−^ mice embryos upon treatment with p53 inhibitor or p53 knockdown [69].

In recent years, however, new studies have reported that Treacle is involved in other cellular functions that may contribute to the development of TCS. In particular, Treacle seems to be involved in DNA damage response (DDR) pathway [70,71]. In NCC cells, the haploinsufficiency of Treacle could also impair the repair of DNA damage caused by high ROS production and activate p53-mediated apoptosis, thus contributing to the development of craniofacial abnormalities in TCS [72]. Nevertheless, despite the important role played by Treacle in DNA damage response, the fact that mutations in Pol I subunits cause symptoms similar to TCOF1 mutations enables us to say that Treacher Collins syndrome mainly develops due to impaired ribosome biogenesis.

## 3. Mixed Ribosomopathies

### 3.1. Dyskeratosis Congenita

On the opposite side of the spectrum of ribosomopathies lies dyskeratosis congenita (DC).

DC is a rare and severe inherited multisystemic syndrome known since the beginning of the last century [73,74,75]. The disorder initially came to the attention of dermatologists because it is characterized by a typical muco-cutaneous triad: abnormal skin pigmentation, dystrophy of the nails, and oral leukoplakia. However, the most severe problem occurring in almost all the DC patients is a progressive bone marrow failure [76]. In addition to these skin and blood defects, one additional relevant aspect of DC is an increased susceptibility to cancer. The overall incidence of malignant tumors in DC patients can reach 50% by the age of 50 [77].

The most frequent form of DC is the X-linked variant, caused by mutations of the *DKC1* gene encoding dyskerin. Dyskerin (aka rat NAP57, Drosophila minifly, yeast Cbf5) is a nucleolar protein endowed with pleiotropic functions, all fundamental to basic cellular events including growth, proliferation, and gene expression control. The functions of dyskerin can be explained considering its ability to bind to H/ACA box snoRNAs [44]. Together with the three other core proteins (namely GAR1, NHP2, and NOP10), dyskerin binds to each snoRNA, guiding the complex to the specific uridine residue for its isomerization to pseudouridine. Most of these modifications occur in ribosomal RNA (rRNA) and small nuclear RNA (snRNA) [78,79,80]. On the other hand, always in association with core proteins, dyskerin binds to the telomerase RNA component (TERC), which also hosts a H/ACA sequence element. This enables TERC stabilization and proper telomerase complex function. DC-associated DKC1 mutations strongly reduce TERC levels and impair telomerase activity [81]. Importantly, other bone marrow failure syndromes classified as DC are due either to mutations of genes encoding for exclusive components of the telomerase complex such as TERC [82] and TERT [83] or to telomere binding proteins [84]. For this reason, most of the clinical features of DC are ascribed to a defect in telomerase function and the disorder is often considered more a telomeropathy than a ribosomopathy [85].

While accepting the fact that the impairment in telomerase activity and the consequent telomere attrition are clearly well-established effects of X-DC-associated DKC1 mutations, this view may, however, appear too simplistic after a careful look at the available evidence. In fact, in all the experimentally generated in vivo models based on *DKC1* gene targeting, a clear defect in the rate of rRNA processing and/or rRNA pseudouridylation was observed [86,87]. In particular, the *Dkc1* hypomorphic mouse recapitulates the signs of DC that have been reported in humans, while *Terc* homozygous deletion in mice induces a clearly much milder phenotype [88]. This indicates that the telomerase-independent effects consequent to a DKC1 defect are sufficient to raise a spectrum of signs consistent with DC in mice. Also, data from DC patients indicate that their cells harbor altered snoRNA regulation and rRNA modification. Although there are forms of DC due to mutations of genes encoding exclusive telomerase complex components or telomere binding proteins, what is reported for X-DC appears to be true also for autosomal recessive forms of DC such as those due to mutations of genes encoding the pseudouridylation core proteins NHP2 and NOP10 [47,48,49]. In addition, DC has been also reported to develop in consequence of *NPM1* gene mutations [50]. In further support of the fact that alterations in rRNA modification are causative of DC, NPM1 mutations found in DC patients cause altered rRNA 2′-*O*-methylation [50]. In summary, the defect in ribosome biogenesis should be considered to contribute, at least to some extent, to the pathogenesis of DC.

### 3.2. Cartilage Hair Hypoplasia-Anauxetic Dysplasia (CHH-AD) Spectrum

Cartilage hair hypoplasia (CHH) is an autosomal recessive inherited disease reported first by Victor McKusick in 1965 in a population of Old Order Amish [89]. This pleiotropic disorder is characterized by short-limbed dwarfism, sparse hypoplastic hair, defective T-cell immunity, hypoplastic anemia, increased risk of developing malignancies [90,91], and other symptoms [92,93,94,95]. Many of these symptoms, including immunodeficiency and cancer predisposition, are considered responsible for a shorter life expectancy in these patients [96]. In 2001, the study by Ridanpaa et al. [8] first described how mutations in the *RMRP* (RNA component of RNase MRP) gene are the molecular cause for CHH. Mutations in this gene are also responsible for other disorders connected with skeletal abnormalities known as the CHH-AD spectrum. Recently a second form of anauxetic dysplasia (AD) not caused by mutations in RMRP but by mutations in POP1, a protein included in the RNase MRP complex [43], has been reported.

RMRP is a long non-coding RNA (lncRNA) that contributes to the formation of the RNase MRP complex with at least seven different proteins, some of which (e.g., POP1) shared with the RNase P complex and implied in tRNA maturation [97]. This complex has many functions in cell nucleus, cytoplasm, and mitochondria that were originally identified in yeast but also described in human cells. In fact, the RMRP complex is an endoribonuclease responsible for the cleavage of mitochondrial RNA, which functions as a primer for mitochondrial DNA replication [98], the cleavage of the 5′UTR of the cyclin B2 mRNA, which leads to a reduction in cyclin B2 synthesis, and cell cycle progression [99]. Recently, viperin (RSAD2) has been described as a novel mRNA substrate for RMRP cleavage [100]. An additional important function of RMRP lncRNA consists of the formation of a complex with the telomerase-associated reverse transcriptase (TERT), which produces a double-stranded RNA with RMRP sequence. These RNA duplexes are cleaved in siRNA by Dicer with the outcome of downregulating the level of RMRP [101]. At this regard, although this latter mechanism does not appear to affect telomerase function, it is worth noting that in two mixed ribosomopathies such as DC and in CHH components of the telomerase complex are involved. Lastly, RMRP is reported to cleave the precursor ribosomal RNA in the ITS1 sequence, leading to the formation of the 5′ end of 5.8S rRNA (as described previously) [102]. This has been confirmed in a recent study in which the disruption of RMRP by CRISPR/Cas9 in HeLa cells led to the accumulation of uncleaved ITS1 [103]. The impairment of this function can cause an altered ribosome biogenesis that makes possible to classify CHH as a ribosomopathy.

Although it is clear that CHH is caused by mutations in the *RMRP* gene, the molecular mechanism underlying the development of the disease is still unclear. A significant number of mutations in the *RMRP* gene have been identified in patients with CHH [104,105], which can involve, in particular, the promoter of *RMRP* or the transcribed region [8]. The first consists of insertions or duplications in the region between the transcription starting site and the TATA box, with the outcome of altering the correct distance between the promoter and the transcription starting site, while reducing the transcription level of *RMRP*. These types of mutations were found mainly in compound heterozygous patients [102], suggesting that at least a minimum level of RMRP is essential for cell life [106]. On the other hand, point mutations or the insertion/deletion of a few nucleotides in the RMRP sequence, in evolutionarily conserved regions important for RNA-protein interaction [107] or for the catalytic activity, result in either a reduced efficiency of the complex or an alteration of RMRP stability [108]. For instance, it has been reported that the most common mutation among the Amish and Finnish patients, but also the most prevalent in the European population, 70A > G, can be found in the putative catalytic pocket, thus reducing the RNase activity [99,109]. In addition, the 70A > G mutation found in CHH patients introduced in the yeast ortholog NME1 resulted in a 5.8Ss/l ratio of 2:3 in comparison with the 10:1 ratio of wild type strains [109].

In the attempt to find a potential causative relationship between the different functions of RMRP and CHH development, it has been reported that RMRP is expressed during the hypertrophic phase of chondrocyte differentiation in mice, and that the trans-differentiation in the chondrocytes from CHH patients’ fibroblasts is impaired in comparison with control fibroblasts. Moreover, CHH fibroblasts show an increase in the ITS1 pre-rRNA processing intermediate, suggesting a reduced ribosome biogenesis [110]. On the other hand, other functions of RMRP can contribute to the development of the pathology. As it was previously described, viperin is a substrate for the endoribonucleolytic activity of RMRP. This protein appears to be involved in the activation of a chondrogenesis regulatory pathway accountable for the reduced chondrocyte differentiation of fibroblasts from CHH patients with mutated RMRP [111]. Moreover, the sequencing analysis of RNA from fibroblasts of CHH patients compared to controls revealed a downregulation in the genes involved in the cell cycle, resulting in a delay in the progression from phase G2 to G1 [112]. Lastly, a recent study reported that the processing of RMRP by Dicer originates two small RNAs with silencing activity on the genes associated with CHH-phenotype [113].

The studies reported on so far highlight the numerous aspects in which RMRP is involved, and all of them can contribute to the pathophysiology of the CHH-AD spectrum: therefore, CHH can rightly be considered a mixed ribosomopathy.

## 4. Recently Identified Ribosomopathies

In recent years, there has been an increase in the number of diseases identified as novel congenital ribosomopathies. These extremely rare diseases are characterized by mutations in ribosomal proteins or in factors involved in ribosome biogenesis, but further studies are necessary to fully understand the contribution of altered ribosome production in their pathophysiology. These ribosomopathies are heterogeneous diseases showing generalized multisystemic symptoms or, alternatively, more specific manifestations selective for one tissue or organ. An example of multisystemic disease is Bowen-Conradi syndrome, a rare autosomal recessive disorder first described by Bowen and Conradi [114] in the Hutterite population, which is characterized by mental retardation, microcephaly, micrognathia, prominent nose, rocker bottom feet, and flexion contractures of the joints [115]. This severe disease is the cause of early death in children; in fact, the average death age is 13 months. Recently, it has been reported that the cause of Bowen-Conradi syndrome is a mutation in the gene *EMG1*, coding for a ribosome assembly protein, thus including this disease in the list of ribosomopathies [116]. The mutation in EMG1 causes 18S rRNA processing delay, with the result of reduced cell proliferation rates and G2/M arrest [117].

A more limited and specific effect is that caused by mutations in a ribosomal protein of the small subunit, RPS20. The mutation in *RPS20* has been associated with a subtype of hereditary colorectal cancer (CRC) called Familial colorectal cancer type X (FCCTX), in which no mutation in mismatch repair genes was reported, but several pathogenic variants of predisposing genes were observed [118,119]. Two different heterozygous mutations of *RPS20* were reported in a CRC-affected family and in a patient with hereditary CRC. The experiment conducted on samples from the CRC-affected family showed that the haploinsufficiency of RPS20 caused a reduced ribosome biogenesis and consequent stabilization of p53, which is probably responsible for the selection of cells that escape p53 regulation [118,119]. Since, for the two examples mentioned above at present, no additional extra-ribosomal functions of the products of the genes involved have been reported, they may be considered putative pure ribosomopathies although further research is necessary to confirm this definition.

A further example of the tissue-specific effect of mutations in ribosomal protein is represented by the outcome of mutations in RPL10. In fact, it has been reported that missense mutations causing an alteration in protein sequence can lead to a rare form of autism [120] or microcephaly [121,122]. A defective nervous system development can be caused by a decreased translational capacity of the cell coupled with an increased apoptosis due to the activation of ribosomal stress response. For RPL10, however, extra-ribosomal functions have also been reported [123]. Ribosomal protein L10 in mitochondria serves as a regulator for the ROS level in pancreatic cancer cells [123,124,125]; therefore, pending a more detailed characterization of the molecular pathogenesis of this disorder, it may be considered a putative mixed ribosomopathy.

In addition to the above-mentioned disorders, other recently identified ribosomopathies have been described. These conditions are listed in Table 2.

## 5. Acquired Ribosomopathies

In addition to inherited ribosomopathies, a defect in the gene encoding for a ribosomal protein is also an underlying factor in an acquired myelodysplastic disorder termed 5q deletion (or 5q−) syndrome. This disorder, which is more frequently found in women over 75 years of age, is due to the somatic deletion of the short arm of chromosome 5, leading to macrocytic anemia and erythroid hypoplasia, which may subsequently progress to AML in some patients [133]. The haploinsufficiency of the *RPS14* gene has been identified by means of an RNA interference-based screening as the predominant cause of the myelodysplastic phenotype in 5q− syndrome [11], indicating that the alteration in the ribosome biogenesis process may also be at the root of acquired disorders. Since for RPS14 no additional extra-ribosomal functions have been reported, 5q− syndrome may be considered a pure acquired ribosomopathy.

It has long been known that the process of ribosome biogenesis is highly deregulated in cancer [12,13], suggesting that a subset of human tumors may also be considered acquired ribosomopathies. Mutations of *NPM1* gene encoding the multifunctional ribosome processing factor nucleophosmin have been described as the most frequent mutation in acute myeloid leukemia [133,134]. Whereas mutations of genes encoding for ribosomal proteins have been reported for the first time in pediatric acute lymphoblastic leukemia, where recurrent mutations of *RPL10* and *RPL5* genes have been found in approximately 10% of all cases [135]. Very interestingly, a large-scale study on more than 10,000 genomes from human tumors of different origins indicated that the hemizygous deletions of ribosomal protein genes occur in more than 40% of the cases [136]. In addition, a growing amount of data has become available on snoRNA mutations and expression alterations in human multiple cancer types [137,138]. All these studies indicate that as ribosome biogenesis deregulation is a frequent feature in cancer. In many cases, cancer itself may be considered, at least to some extent, an acquired ribosomopathy. The exact role of most of these ribosome biogenesis alterations, however, still remains to be determined.

A list of acquired ribosomopathies is also shown in Table 3.

## Figures and Tables

**Table 1:** Congenital/Inherited ribosomopathies.

Congenital/Inherited Ribosomopathies				
Disease	Gene Mutated	Role in Ribosome Biogenesis	Clinical Manifestations	Type of Ribosomopathy
Diamond Blackfan anemia	*RPS19*, *RPS26*, *RPS17*, *RPS29* [30] *RPS28* [31] *RPS24*, *RPL5*, *RPL11* [32] *RPL35*, *RPL18* [33] *RPL26* [34] *RPL15* [35] *RPS27*, *RPL27* [36]	40S and 60S subunits protein	Macrocytic anemia, skeletal abnormalities, short stature, cardiac and genitourinary malformations, cancer predisposition	Pure
Shwachman-Diamond syndrome	*SBDS* [7] *DNAJC21* [37] *EFL1* [38] *SRP54* [39]	Assembly of 60S and 40S subunits in active 80S ribosomes	Bone marrow failure, skeletal dysplasia, cognitive impairment, and risk of developing myelodysplastic syndrome	Pure
Treacher Collins syndrome	*TCOF1* [40] *POLR1C*, *POLR1D* [41] *POLR1B* [42]	Ribosomal RNA transcription	Severe craniofacial defects and mental retardation	Pure
Cartilage Hair Hypoplasia-Anauxetic dysplasia spectrum	*RMRP* [8] *POP1* [43]	Ribosomal RNA processing	Short-limbed dwarfism, sparse hypoplastic hair, immunodeficiency, hypoplastic anemia, and predisposition to cancer	Mixed
Dyskeratosis Congenita	*DKC1* [4,44] *PARN* [45,46] *NHP2*, *NOP10* [47,48,49] *NPM1* [50]	Ribosomal RNA pseudouridylation and processing	Abnormal skin pigmentation, dystrophy of the nails, oral leukoplakia, bone marrow failure, and cancer predisposition	Mixed

The present review aims to classify the disorders due to a defect in ribosome production, highlighting their features as pure or mixed ribosomopathies.

**Table 2:** Recently identified ribosomopathies.

Recently Identified Ribosomopathies			
Disease	Gene Mutated	Role in Ribosome Biogenesis	Clinical Manifestations
Alopecia, neurologic defects, and endocrinopathy syndrome	*RBM28* [126]	Ribosomal RNA processing	Alopecia, mental retardation, progressive motor deterioration, central hypogonadotropic hypogonadism and short stature, microcephaly, gynecomastia, and hypodontia
North American Indian Childhood Cirrhosis	*CIRHIN* [127] *NOL11* [128]	18S rRNA processing	Transient neonatal jaundice that evolves into biliary cirrhosis requiring hepatic transplantation
Bowen-Conradi syndrome	*EMG1* [116]	Ribosome assembly	Mental retardation, microcephaly, micrognathia, rocker bottom feet, and flexion contractures of the joints; causes early death
Familial colorectal cancer type X	*RPS20* [118]	40S subunit protein	Hereditary colorectal cancer without mutations in mismatch repair genes
Congenital asplenia	*RPSA* [129]	40S subunit protein	Absence of spleen
Aplasia cutis congenita	*BMS1* [130]	Ribosomal GTPase, 18S rRNA processing	Skin defect and alopecia of the scalp
RPS23-related ribosomopathy	*RPS23* [131]	40S subunit protein	Microcephaly, hearing loss, dysmorphic features, intellectual disability, and autism spectrum disorder
Leukoencephalopathy, intracranial calcifications, and cysts (LCC)	*SNORD118* [132]	C/D box snoRNA U8 involved in ribosome biogenesis	Neurological disorder with leukoencephalopathy, intracranial calcifications, and cysts
Autism	*RPL10* [120]	60S subunit protein	Autism spectrum disorder
Microcephaly	*RPL10* [121,122]	60S subunit protein	Microcephaly, intellectual disability, epilepsy, and growth retardation

**Table 3:** Acquired ribosomopathies.

Acquired Ribosomopathies			
Disease	Gene Mutated	Role in Ribosome Biogenesis	Clinical Manifestations
5q− syndrome	*RPS14* [11]	40S subunit protein	Macrocytic anemia and erythroid hypoplasia; may progress to AML
Acute myeloid leukemia (AML)	*NPM1* [139]	Ribosome processing	AML with normal karyotype
Pediatric acute lymphoblastic leukemia (T-ALL)	*RPL5*, *RPL10*, *RPL22* [135]	60S subunit proteins	T-ALL
Relapsed CLL	*RPS15*, *RPSA*, *RPS20* [140]	40S subunit proteins	Relapse after first-line treatment
